# Visual Food Ingredient Prediction Using Deep Learning with Direct F-Score Optimization

**DOI:** 10.3390/foods14244269

**Published:** 2025-12-11

**Authors:** Nawanol Theera-Ampornpunt, Panisa Treepong

**Affiliations:** College of Computing, Prince of Songkla University, Phuket 83120, Thailand; nawanol.t@phuket.psu.ac.th

**Keywords:** F-score optimization, loss function, deep learning, food ingredient prediction, image classification, cost-sensitive learning

## Abstract

Food ingredient prediction from images is a challenging multi-label classification task with significant applications in dietary assessment and automated recipe recommendation systems. This task is particularly difficult due to highly imbalanced classes in real-world datasets, where most ingredients appear infrequently while several common ingredients dominate. In such imbalanced scenarios, the F-score metric is often used to provide a balanced evaluation measure. However, existing methods for training artificial neural networks to directly optimize for the F-score typically rely on computationally expensive hyperparameter optimization. This paper presents a novel approach for direct F-score optimization by reformulating the problem as cost-sensitive classifier optimization. We propose a computationally efficient algorithm for estimating the optimal relative cost parameters. When evaluated on the Recipe1M dataset, our approach achieved a micro F1 score of 0.5616. This represents a substantial improvement from the state-of-the-art method’s score of 0.4927. Our F-score optimization framework offers a principled and generalizable solution to class imbalance problems. It can be extended to other imbalanced binary and multi-label classification tasks beyond food analysis.

## 1. Introduction

Automated food analysis has emerged as a pivotal area of research with diverse applications. These include nutrition monitoring [1,2,3,4], culinary recommendation systems [5,6,7,8], food quality classification [9,10,11], and culinary culture analysis [12,13,14]. The ability to accurately predict food ingredients from visual information alone represents a fundamental challenge in computer vision. Models need to capture complex relationships between visual appearance and the underlying ingredients. This task is particularly challenging for several reasons. First, there is inherent variability in food presentation and preparation methods. Second, the visual cues that distinguish different ingredients within prepared dishes are often subtle. Third, many ingredients are not visible once the food is prepared. Therefore, the model needs to infer hidden ingredients based on visible elements.

Traditional approaches to automated food analysis have primarily focused on food recognition and classification tasks. In these tasks, models are trained to identify specific dishes or food categories [15,16,17]. However, ingredient prediction is a more fine-grained task that presents greater challenges. It requires understanding the composition of foods rather than merely recognizing their overall appearance. This multi-label classification problem is further complicated by the imbalanced nature of ingredient distributions in real-world datasets. Common ingredients appear frequently while specialized or regional ingredients occur rarely [18].

Recent advances in deep learning, particularly convolutional neural networks (CNNs) and vision transformers (ViTs) [19], have demonstrated remarkable success in various computer vision tasks [20,21,22]. These architectures have also shown promise in food-related applications. CNNs excel at capturing local textural and spatial features that are crucial for identifying food components. In contrast, ViTs offer superior performance in modeling long-range dependencies and global context understanding [23,24,25]. However, when these models are applied to ingredient prediction with F-score as the main metric, their potential has been limited. This limitation stems from the the lack of optimization methods specifically designed for the F-score.

The F-score is the harmonic mean of precision (Pr) and recall (Rc). It provides a balanced measure of model performance and is particularly relevant for scenarios where the classes are highly imbalanced, such as the ingredient prediction task. The standard cross-entropy loss allows for the optimization of accuracy when classes are unweighted and for balanced accuracy when classes are weighted inversely proportional to class frequencies. However, both of these approaches lead to low F-scores. Existing approaches to adjusting the cross-entropy loss for direct F-score optimization are limited. One traditional approach is class weight hyperparameter tuning, which is time-consuming. Another approach is post hoc threshold tuning to maximize F-score, which leads to suboptimal performance.

This paper addresses these limitations by proposing a novel approach for direct F-score optimization. We show that F-score optimization can be reduced to cost-sensitive classification. Our formulation can be applied to the binary cross-entropy (BCE) loss and other binary loss functions. Therefore, it can be utilized in any task and any model with binary output(s). We leveraged standard CNN and ViT architectures to extract visual features. We demonstrate that our approach leads to significant improvements in food ingredient prediction accuracy.

The contributions of this work can be summarized as follows:

1. We present a novel cost-sensitive formulation for direct F-score optimization. This formulation can be utilized in both single and multiple output scenarios, and for both micro and macro F-scores.
2. We provide a practical approximation algorithm that enables deep learning model training without additional computation.
3. We conducted comprehensive experiments on the Recipe1M dataset comparing CNN and ViT architectures for visual food ingredient prediction. These experiments provide insights into their relative strengths and limitations.
4. We demonstrated that our method offers substantial improvements over alternative F-score optimization approaches. These alternatives include searching for the optimal relative misclassification cost and soft $F_{\beta }$ loss. Our method also outperforms existing state-of-the-art methods on food ingredient prediction.

This newly established benchmark for visual food ingredient prediction has practical implications for multiple applications. These include dietary monitoring, automated recipe generation systems, and culinary recommendation systems. In these applications, accurate ingredient identification is crucial for providing reliable recommendations and assessments.

## 2. Related Work

### 2.1. Food Ingredient Prediction

Food computing [26] has emerged as a rapidly growing research field. This growth is driven by the widespread availability of food-related data and increasing awareness of nutrition’s critical role in health outcomes. Within this domain, several tasks have been formulated. Food recognition and calorie estimation are the most closely related to food ingredient prediction.

Food recognition focuses on identifying dish names from images. Bossard et al. [27] created Food-101, an early comprehensive food recognition dataset containing 101,000 images across 101 classes. The authors proposed a classification method based on random forests and AlexNet. This dataset has since become a standard benchmark. It has spurred numerous improvements in models and training techniques [28,29,30,31,32,33,34,35]. More recent datasets focusing on single cuisines [36,37,38,39] and multiple cuisines [40,41,42,43] have further enriched the evaluation landscape for food recognition methods.

Food calorie estimation typically involves three sequential steps: food recognition or segmentation, volume estimation, and calorie density database lookup. Volume estimation methods vary in their requirements. Some utilize single-view approaches [44,45,46] while others use dual-view approaches [47,48]. These methods often require reference objects or camera calibration. They assume typical ingredient compositions and quantities when estimating calories. Food ingredient prediction extends beyond this by identifying both visible and non-visible ingredients from food images.

Several recent studies have specifically addressed ingredient prediction. Chen and Ngo [49] proposed a deep learning approach for ingredient recognition in cooking recipe retrieval. The CNN architecture VGG-16 was employed for visible ingredient recognition. Conditional random fields were used to refine predictions based on ingredient relationships. The method achieved a 0.67 micro F1 score on a Chinese food dataset with 110,241 images and 353 ingredients. However, this work was limited to directly visible ingredients. In contrast, our approach predicts all ingredients regardless of visibility.

Liu et al. [50] utilized an attention fusion network paired with balanced focal loss for ingredient recognition. The authors addressed class imbalance through unbalanced misclassification costs based on the square root of relative class frequencies. Our work demonstrates that this represents a suboptimal cost assignment that prevents models from reaching their full potential.

Two notable studies have addressed recipe generation from food images, incorporating ingredient prediction as a key component. Salvador et al. [51] proposed a multi-component architecture. It consisted of an image encoder, an ingredient decoder, an ingredient encoder, and an instruction decoder. Their approach included specialized loss functions to improve ingredient cardinality estimation. The method achieved an F1 score of 0.4826 on the Recipe1M dataset. Chhikara et al. [52] employed ViT as visual feature extractors with transformer-based ingredient decoders. They achieved a slightly improved F1 score of 0.4927. Both studies serve as important baselines for our comparative evaluation. We demonstrate superior performance despite using comparable or smaller image encoder architectures.

### 2.2. F-Score Optimization

Early research on F-score optimization in machine learning primarily focused on classical methods. These included logistic regression [53,54,55], support vector machines [56,57], and specialized algorithms [58,59,60,61]. However, these approaches cannot be directly applied to neural network optimization, which relies on more complex stochastic gradient descent (SGD) algorithms.

One line of research has investigated optimal thresholding strategies for classifiers [62,63,64,65]. While these methods apply to broad classes of classifiers, they cannot guide the model optimization process toward improved data point ranking capabilities.

Pastor-Pellicer et al. [66] proposed using F-score directly as a loss function. The method replaces true positives (TP), false positives (FP), and false negatives (FN) with soft counts. When training with mini-batches, only soft counts from the current batch are used. This method can potentially lead to high gradient variance. It may also produce undefined gradients when there are no positive predictions, leading to inferior results. Additionally, the authors used training set counts. These counts likely provide insufficient weight to positive classes. This is because metrics typically exhibit higher values on training sets compared to validation sets. Our approach solves both of these issues by using the current best estimates of TP, FP, and FN counts based on the validation set.

Puthiya Parambath et al. [67] proposed optimizing F-measures through reduction to cost-sensitive classification. The relative costs were given in terms of optimal F-measures. Since optimal F-measures are generally unknown, the authors suggested an approximation algorithm that tests multiple values. While conceptually similar to our approach, their work lacks derivation of the relative costs’ theoretical foundation. In contrast, we provide a straightforward derivation of relative costs from the objective function. In addition, we propose a more efficient practical algorithm. Our algorithm provides an approximation of the optimal relative cost without incurring additional computation. Their method was evaluated using support vector machines and logistic regression on tabular and text datasets. In contrast, we demonstrate our method’s effectiveness on neural networks using image data.

Eban et al. [68] proposed a general framework for optimizing non-decomposable objective functions. The framework uses surrogate functions such as hinge loss as bounds for the true objective. For F-score optimization, they reduced the problem to cost-sensitive classification similar to Puthiya Parambath et al. [67]. The same issues as the former work exist, and no practical approximation algorithm was provided. Their evaluation focused on the area under the precision-recall curve (AUCPR) metric rather than F-score.

Our formulation provides several advantages over existing approaches. First, it offers transparent theoretical derivation of cost parameters. Second, it provides a computationally efficient practical algorithm that does not require additional hyperparameter tuning. Third, we conduct evaluation on deep learning architectures using real image datasets. Consequently, our method is particularly suitable for large-scale models and datasets.

## 3. Materials and Methods

### 3.1. Model Architecture

**Figure 1:**
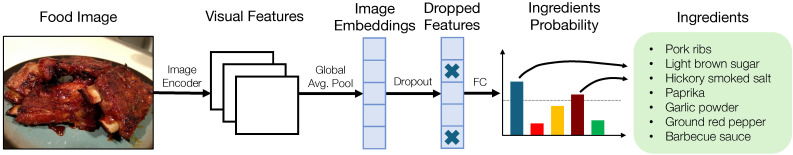
Overview of our visual food ingredient prediction architecture. Each bar in the bar chart corresponds to a single ingredient.

The architecture of our models is illustrated in Figure 1. A preprocessed food image is provided as input to the model. A deep learning-based image encoder backbone, such as a CNN or ViT, extracts visual features from the image. The features are pooled using global average pooling. The pooled features are flattened to produce a vector of image embeddings. A dropout layer is incorporated to reduce overfitting. Finally, the image embeddings are used to predict ingredients through a fully connected (FC) output layer with one binary node per ingredient. Ingredients with predicted probabilities of at least 0.5 are included in the final predicted ingredient set.

Any deep learning backbone can serve as the image encoder. For our work, we utilize small and efficient CNNs and ViTs to maintain system practicality for mobile device deployment. These backbone architectures are detailed in Table 1. All of these architectures can make single-image inferences using 224×224 images with less than 1 s latency on smartphone CPUs [69].

Our architecture treats predicted ingredients as a set. Ingredients are conditionally independent given the image embeddings. Although explicit modeling of ingredient dependencies (e.g., the co-occurrence of *flour* and *butter*) is not incorporated, ingredient prediction based on shared image embeddings can effectively capture these relationships. For example, suppose that *flour* is a visible ingredient and *butter* is not a visible ingredient, but they are frequently used together. The model learns from training data that bread-like textures are associated with *butter*. When the model sees an image with bread-like textures, it predicts that *butter* is used. In fact, the model may never know (and does not need to know) what *butter* looks like before cooking at all. This is how the model can infer non-visible ingredients from visible elements.

We do not employ explicit ingredient set cardinality loss functions, as the F-score already inherently penalizes both overprediction and underprediction. In addition, correct cardinality is meaningless if the predicted ingredients are wrong. While probability thresholds for ingredient prediction could be further tuned after model training, we argue that a good model optimization process should produce models that perform well with predetermined thresholds. Therefore, we use a fixed threshold of 0.5. An ingredient is predicted only when its probability meets or exceeds this threshold.

### 3.2. F-Score Optimization

#### 3.2.1. Motivation

The F-score (or F-measure) is typically employed as the primary evaluation metric for binary classification tasks where the focus is on the positive class. However, to directly optimize for F-score, it remains unclear which loss function should be used and how the class weights should be set during model training. In this work, we derive the optimal class weights for direct F-score optimization. We also provide a practical algorithm for neural network training using this approach.

The standard loss function for binary classification is the BCE loss, defined as:

$$\begin{array}{cc} \ell (x,y) & =-w[y\cdot \log x+(1-y)\cdot \log(1-x)] \end{array}$$

where *w* is the weight assigned to the data point, *x* is the predicted probability of the positive class, and *y* is the ground truth class label. The BCE loss has desirable properties mentioned in Section 3.2.5 and strong theoretical guarantees, making it the most commonly used loss function in binary classification tasks [76].

With equal data point weights, this loss function optimizes accuracy. In cases of unbalanced classes, the model focuses disproportionately on the majority class, resulting in low Rc and F-score. To address class imbalance, two weight values are employed: $w_{\mathrm{pos}}$ and $w_{\mathrm{neg}}$ for positive and negative classes, respectively. These values are referred to as class weights. Since only the ratio of these class weights matters, we set $w_{\mathrm{neg}}=1$ without loss of generality.

A common approach sets $w_{\mathrm{pos}}$$=\frac{N_{\mathrm{neg}}}{N_{\mathrm{pos}}}$, where $N_{\mathrm{neg}}$ and $N_{\mathrm{pos}}$ are the numbers of negative and positive samples, respectively. This approach ensures equal sum of weights across classes and optimizes balanced accuracy (the average of true positive rate and true negative rate). However, when the positive class is in the minority, high balanced accuracy often leads to low F-score due to high Rc but low Pr. The optimal $w_{\mathrm{pos}}$ therefore lies within the range $\left(1,\frac{N_{\mathrm{neg}}}{N_{\mathrm{pos}}}\right)$. Finding this optimal value traditionally requires trial and error, complicating the training process. We will derive the optimal $w_{\mathrm{pos}}$ directly from the objective function.

#### 3.2.2. Optimal $w_{\mathrm{pos}}$ Derivation

Our objective function is the $F_{\beta }$ score, defined as:

(1) $$\begin{array}{c} F_{\beta }=\frac{(1+\beta^{2})\cdot \mathrm{Pr}\cdot \mathrm{Rc}}{(\beta^{2}\cdot \mathrm{Pr})+\mathrm{Rc}} \end{array}$$

for β∈(0,∞). Pr is defined as:

(2) $$\begin{array}{cc} \mathrm{Pr} & =\frac{\mathrm{TP}}{\mathrm{TP}+\mathrm{FP}} \end{array}$$

and Rc is defined as:

(3) $$\begin{array}{cc} \mathrm{Rc} & =\frac{\mathrm{TP}}{\mathrm{TP}+\mathrm{FN}} \end{array}$$

Substituting Equations (2) and (3) into Equation (1), we obtain:

(4) $$\begin{array}{cc} F_{\beta } & =\frac{\left(1+\beta^{2}\right)\cdot \frac{\mathrm{TP}}{\mathrm{TP}+\mathrm{FP}}\cdot \frac{\mathrm{TP}}{\mathrm{TP}+\mathrm{FN}}}{\left(\beta^{2}\cdot \frac{\mathrm{TP}}{\mathrm{TP}+\mathrm{FP}}\right)+\frac{\mathrm{TP}}{\mathrm{TP}+\mathrm{FN}}}\\ \; & =\frac{(1+\beta^{2})\cdot \mathrm{TP}}{\left(1+\beta^{2}\right)\cdot \mathrm{TP}+\beta^{2}\cdot \mathrm{FN}+\mathrm{FP}} \end{array}$$

To eliminate the interdependence between FN and TP, we substitute $\mathrm{TP}=N_{\mathrm{pos}}-\mathrm{FN}$ in Equation (4):

(5) $$\begin{array}{cc} F_{\beta } & =\frac{\left(1+\beta^{2}\right)\cdot \left(N_{\mathrm{pos}}-\mathrm{FN}\right)}{\left(1+\beta^{2}\right)\cdot \left(N_{\mathrm{pos}}-\mathrm{FN}\right)+\beta^{2}\cdot \mathrm{FN}+\mathrm{FP}}\\ \; & =\frac{\left(1+\beta^{2}\right)\cdot \left(N_{\mathrm{pos}}-\mathrm{FN}\right)}{\left(1+\beta^{2}\right)\cdot N_{\mathrm{pos}}-\mathrm{FN}+\mathrm{FP}} \end{array}$$

From Equation (5), we compute the partial derivatives of $F_{\beta }$ with respect to FP and FN:

(6) $$\begin{array}{c} \frac{\partial F_{\beta }}{\partial \mathrm{FP}}=-\frac{\left(1+\beta^{2}\right)\cdot \left(N_{\mathrm{pos}}-\mathrm{FN}\right)}{{\left(\left(1+\beta^{2}\right)\cdot N_{\mathrm{pos}}-\mathrm{FN}+\mathrm{FP}\right)}^{2}} \end{array}$$

(7) $$\begin{array}{c} \frac{\partial F_{\beta }}{\partial \mathrm{FN}}=-\frac{\left(1+\beta^{2}\right)\cdot \left(\beta^{2}\cdot N_{\mathrm{pos}}+\mathrm{FP}\right)}{{\left(\left(1+\beta^{2}\right)\cdot N_{\mathrm{pos}}-\mathrm{FN}+\mathrm{FP}\right)}^{2}} \end{array}$$

Since $w_{\mathrm{pos}}$ represents the relative penalty of misclassifying a positive sample (FN) compared to misclassifying a negative sample (FP), we compute $w_{\mathrm{pos}}$ by dividing Equation (7) by Equation (6):

(8) $$\begin{array}{cc} w_{\mathrm{pos}} & =\frac{\beta^{2}\cdot N_{\mathrm{pos}}+\mathrm{FP}}{N_{\mathrm{pos}}-\mathrm{FN}}\\ \; & =\frac{\beta^{2}\cdot \mathrm{TP}+\beta^{2}\cdot \mathrm{FN}+\mathrm{FP}}{\mathrm{TP}}\\ \; & =\frac{\mathrm{TP}+\mathrm{FP}}{\mathrm{TP}}+\frac{\beta^{2}\cdot \left(\mathrm{TP}+\mathrm{FN}\right)}{\mathrm{TP}}-1\\ \; & =\frac{1}{\mathrm{Pr}}+\frac{\beta^{2}}{\mathrm{Rc}}-1 \end{array}$$

Given that we are optimizing for the F-score on the test set, Pr and Rc in Equation (8) must be the test set metrics from the globally optimal model.

#### 3.2.3. Practical Algorithm

We need the globally optimal model to find Pr and Rc in Equation (8). However, if the globally optimal model is already found, there would be no need to optimize the model further. We propose a practical algorithm and heuristics as a solution to this chicken-and-egg problem.

The task at hand is to estimate Pr and Rc of the optimal model without having the optimal model. Several approaches exist for estimating these metrics. If reliable estimates of final Pr and Rc are available (e.g., from previous training runs, prior work, or domain expertise), they can be used directly to compute $w_{\mathrm{pos}}$. In this case, $w_{\mathrm{pos}}$ should be kept constant throughout the training process.

When such estimates are unavailable, we propose Fola, a practical algorithm for training neural networks to maximize F-score. The algorithm is illustrated in Algorithm 1.
**Algorithm 1** Fola: Training neural network to maximize $F_{\beta }$-score1:**procedure** Train(model, train_data, val_data, β, $w_{\mathrm{pos}}$, num_epochs)2:    **for** epoch=1,…,num_epochs **do**3:        $loss\_fn=\mathrm{BCE}(w_{\mathrm{pos}})$4:        train_epoch(model,train_data,loss_fn)5:        Pr, Rc← evaluate_epoch(model,val_data)6:        $w_{\mathrm{pos}}\leftarrow 1/Pr+\beta^{2}/Rc-1$7:    **end for**8:**end procedure**

The main idea is to start with an initial estimate of $w_{\mathrm{pos}}$, then update and use it during the model training process. The initial $w_{\mathrm{pos}}$ value is user-specified. When no estimate is available, the upper bound $w_{\mathrm{pos}}$$=\frac{N_{\mathrm{neg}}}{N_{\mathrm{pos}}}$ serves as a reasonable starting point. At each training epoch, we construct a BCE loss function with the specified $w_{\mathrm{pos}}$. We train the model for one epoch using this loss function and evaluate it on validation data. The Pr and Rc metrics from validation data are then used to compute an updated estimate for $w_{\mathrm{pos}}$. Training continues until the specified number of epochs is completed. When $w_{\mathrm{pos}}$ becomes undefined due to zero TP, we revert to the user-specified initial value.

The Fola algorithm works because after every epoch, the estimates of the optimal Pr and Rc become closer to the true optimal values. These values are used to compute a better estimate of $w_{\mathrm{pos}}$, and so on. Deep learning models typically attain most of their final accuracy within the first few epochs. Therefore, a reasonably accurate estimate of the optimal $w_{\mathrm{pos}}$ can be obtained early in the training process.

At each epoch, the current Pr and Rc metrics from the previous epoch represent the best available estimates. Crucially, we use validation set metrics rather than training set metrics. Using the latter would underestimate $w_{\mathrm{pos}}$ due to models typically achieving better performance on training data. The initial $w_{\mathrm{pos}}$ can be computed from human-provided estimates or other heuristics. Poor initial values are unlikely to significantly impact final results because better validation-based estimates are used from the second epoch onward.

In this work, we employed cyclical learning rates (CLR) [77] to train our models. CLR is a learning rate (LR) schedule where LRs start low and gradually increase. For other schedules that begin with high LRs (e.g., cosine decay and exponential decay), we recommend a linear warmup period of at least two epochs to prevent poor initial $w_{\mathrm{pos}}$ values from disrupting training.

For more accurate $w_{\mathrm{pos}}$ estimates, model training can be repeated multiple times. In each subsequent run, $w_{\mathrm{pos}}$ is computed from the previous run’s final results and kept fixed throughout training. When changes in $w_{\mathrm{pos}}$ become sufficiently small, convergence is achieved.

During test set evaluation, $w_{\mathrm{pos}}$ cannot be computed from test predictions, as this would constitute information leakage from the test set. Instead, we use final Pr and Rc from the validation set to compute $w_{\mathrm{pos}}$ and maintain this value throughout training.

The Fola algorithm applies to both binary classification and multi-label classification with binary outputs. For multi-label tasks, it can optimize both micro and macro F-scores. For micro F-scores, TP, FN, and FP counts are pooled across labels, allowing for direct application of the algorithm. For macro F-scores, counts are maintained separately for each label to compute individual F-scores. Since individual F-score contributions to the overall score are known, we apply the BCE loss separately to each label, with different $w_{\mathrm{pos}}$ values based on each label’s Pr and Rc.

Our derived $w_{\mathrm{pos}}$ does not apply to multi-class F-score optimization. However, we believe our derivation method and the Fola algorithm can be applied to multi-class F-score optimization, but this is outside the scope of this work. Nevertheless, we argue that F-score loses its main characteristic of focusing on a single class in multi-class scenarios and thus there is no good reason to use it. We instead recommend either grouping classes into binary categories before applying F-score, or using alternative metrics. Accuracy and balanced accuracy are standard metrics in such cases, since no single class can be designated as the focal positive class.

#### 3.2.4. Considerations for Macro F-Score Optimization

While the derived $w_{\mathrm{pos}}$ can be straightforwardly applied to macro F-score optimization in multi-label binary classification, we have empirically observed suboptimal results in some cases. In multi-label classification, separate loss functions are used for each label. Weight updates computed from each loss function are applied consecutively. However, $w_{\mathrm{pos}}$ values across labels can vary dramatically. This leads to overshooting during weight updates for some labels, which degraded overall prediction accuracy. Scaling down the LRs for all labels would solve this problem but would introduce another problem of slow convergence. To keep our approach practical, we bound $w_{\mathrm{pos}}$ values at twice the median $w_{\mathrm{pos}}$ value. Low $w_{\mathrm{pos}}$ values do not present similar problems and can remain unchanged. This bounding factor is an additional hyperparameter. If computational efficiency is prioritized, it can be fixed at 2, as we have found that this value works well in practice. If prediction accuracy is prioritized, it can be tuned to find the optimal value for the task.

#### 3.2.5. Alternative Approach

A straightforward alternative to our method involves using F-score directly as the loss function. Since model training is formulated as loss minimization while higher F-scores are preferable, we multiply F-score by −1. To ensure differentiability, soft counts are employed: TP=O·T, FP=O·(1−T), and FN=(1−O)·T, where *O* represents the model’s probability of positive class outputs and *T* represents true labels (0 for negative class and 1 for positive class). We refer to this approach as soft $F_{\beta }$ loss and demonstrate that our method achieves superior performance.

The soft $F_{\beta }$ loss is equivalent to using $w_{\mathrm{pos}}$ with the mean absolute error (MAE) loss instead of BCE loss. An important property of the BCE loss is that when the output is uncertain, the optimal output probability is exactly the true probability distribution. For example, when the model is 60% certain that the output is positive, the output with the lowest loss would be to predict positive with 60% probability. On the other hand, the optimal output for the MAE loss is the median, which in the context of a binary task is the class with higher probability. In this case, the model would predict positive with 100% probability. Forcing the model to be confident when it is not leads to inferior results. Theoretical analysis of guarantees provided by cross-entropy loss is given in [76].

## 4. Results

In this section, we describe the dataset and experimental setup, and present and discuss the results. We first compared deep learning architectures for visual food ingredient prediction. Then, we evaluated our proposed optimization framework against other loss functions and alternatives. Separate experiments were conducted for micro and macro F1 scores to assess classification performance in both scenarios.

### 4.1. Dataset

We utilized the Recipe1M dataset [18] to train and evaluate all models. Recipe1M contains over 1 million recipes and 800,000 food images collected from cooking websites. Each recipe is associated with zero or more images. Recipes without associated images were removed as they cannot be used for our visual prediction task. We extracted ingredient names from ingredient lists using the same procedure as Salvador et al. [51] to enable direct comparison of results.

**Figure 2:**
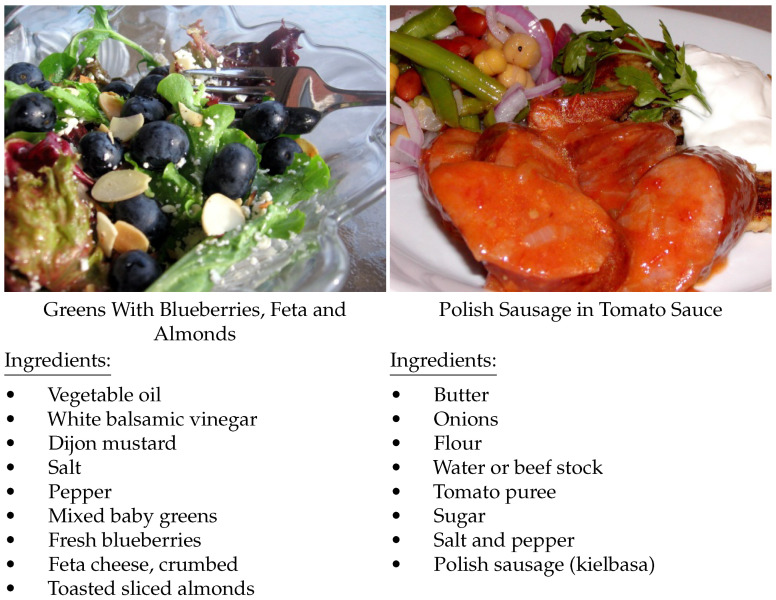
Examples of Recipe1M data. We only used the images and ingredient lists.

During this procedure, ingredients within the same category (e.g., various types of *cheese*) were grouped together through a three-step process. The grouping procedure involved: (1) merging ingredients with identical first or last two words (e.g., merging *bacon cheddar cheese* with *cheddar cheese*), (2) clustering ingredients with matching words in either the first or last position (e.g., *gorgonzola cheese* and *cheese blend*), and (3) combining different forms of the same word, including singular/plural variations and misspellings. Ingredients that appeared in fewer than ten recipes were removed. Only recipes containing 2–20 ingredients were included. After filtering, the dataset contained 1488 unique ingredients and 819,055 images. Dish names and cooking instructions were not utilized. The train/validation/test splits followed an approximate 70%/15%/15% distribution. Figure 2 depicts examples of data in Recipe1M.

**Figure 3:**
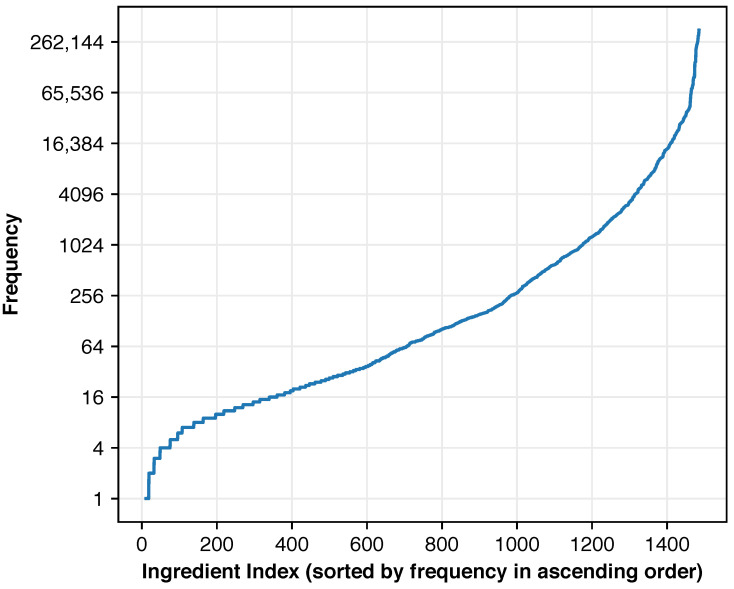
Distribution of ingredient frequencies in the Recipe1M dataset, illustrating the extreme class imbalance between different ingredients. Frequency denotes the number of food images prepared using each ingredient.

The top ingredients in the dataset are listed in Table 2, with ingredient frequency distributions illustrated in Figure 3. Frequency represents the number of food images prepared using each ingredient. The distribution exhibited severe imbalance, with the top 16 ingredients accounting for half of all occurrences. The overall negative-to-positive label ratio was 185:1, highlighting the challenging nature of this multi-label classification task.

### 4.2. Implementation Details

All images were center-cropped to square dimensions and resized to 224×224 pixels. Preprocessing, model training, and evaluation were implemented in Python 3.12.3 using PyTorch 2.9.0 [78]. All models were initialized with ImageNet-1K [79] pretrained weights at 224×224 resolution. The models were then adapted to our dataset using transfer learning by modifying the output layer to 1488 nodes. A global average pooling layer and dropout layer were added before the final output layer. Model training consists of separate transfer learning fine-tuning phases.

Experiments were conducted on a single NVIDIA GeForce RTX 5090 graphics processing unit with 32 gigabytes of memory, manufactured by Micro-Star International and sourced in Phuket, Thailand. Hyperparameter values are detailed in Table 3. LRs were optimized separately for each experimental run. Data augmentation was limited to random horizontal flips during training only. For micro F1 score optimization, we set initial $w_{\mathrm{pos}}$ to 3, based on expected Pr and Rc values of 0.5 from previous work [51,52]. For macro F1 score optimization, we initialized $w_{\mathrm{pos}}$ for each ingredient to $\frac{N_{\mathrm{neg}}}{N_{\mathrm{pos}}}$ for that ingredient, as no prior baselines existed. We did not perform multiple training iterations to readjust $w_{\mathrm{pos}}$, as single-pass optimization proved sufficient. Training runs for each configuration were repeated three times in total. We report the average metric values along with the standard deviations. We performed statistical significance tests using one-tailed unpaired Student’s *t*-tests with unequal variances.

### 4.3. Baselines

We compared our approach against the following baseline methods:

- **InverseCooking** [51] employs ResNet50 as the image encoder and either a set transformer (denoted ${\mathrm{TF}}_{set}$) or feedforward network (denoted ${\mathrm{FF}}_{TD}$) as the ingredient decoder. We used the set transformer variant as the primary baseline due to its superior performance.
- **FIRE** [52] utilizes ViT [19] as the image encoder with a set transformer ingredient decoder, similarly to InverseCooking’s architecture.
- **BCE w/ fixed $w_{\mathrm{pos}}$** uses a fixed value for the $w_{\mathrm{pos}}$ parameter of the BCE loss function. We evaluated multiple values to demonstrate the impact of suboptimal class weight selection.
- **Soft $F_{\beta }$** employs a differentiable version of F-score as the loss function directly by replacing hard counts with sums of predicted probability. This approach does not rely on auxiliary loss functions such as BCE.

### 4.4. Model Comparison for Micro F1 Score

#### 4.4.1. Overall Prediction Performance

We compared our Fola optimization algorithm with prior work on visual food ingredient prediction. Six deep learning architectures were used: MaxViT-T, SwinV2-T, EfficientNetV2-S, RegNetY-1.6GF, ResNet-50, and EfficientViT-M5. Models were evaluated using intersection over union (IoU) and micro F1 score. IoU of a data point is the size of the intersection divided by the size of the union of the true and predicted sets of ingredients. IoU is computed for each data point separately before averaging. On the other hand, F1 score is computed for all data points of each ingredient separately before combining. Therefore, IoU is row-based, while F1 score is column-based. We included both metrics so that we can view the results in both dimensions and to enable direct comparison between our approach and prior work. The micro F1 score uses cumulative TP, FN, and FP counts across all ingredients, effectively weighting each ingredient proportionally to its frequency. Results are presented in Table 4.

MaxViT-T achieved the highest performance with an IoU of 0.4014 and F1 score of 0.5616, followed closely by SwinV2-T and EfficientNetV2-S. The differences in F1 scores between MaxViT-T and the other five models were statistically significant (*p*-value ≤0.0384). Performance differences among the top four models were modest. However, ResNet-50 and EfficientViT-M5 showed notably lower performance. Importantly, all six models combined with Fola significantly outperformed both previous studies. InverseCooking used ResNet-50 as its backbone, while **FIRE** employed the larger ViT [19] model. Based on this direct comparison, we attribute the accuracy improvements to the Fola algorithm and our overall prediction architecture (an ablation study is presented in Section 4.5).

The relatively modest F1 scores across all approaches reflect the inherent difficulty of the task. Predicting ingredients from images alone requires deep understanding of how cooking processes and ingredient combinations alter visual appearance, as well as knowledge of culinary norms and ingredient dependencies. The extreme class imbalance ratio of 185:1 further compounded these challenges.

#### 4.4.2. Cardinality Analysis

Table 5 presents cardinality analysis results for predicted ingredient sets. ${\mathrm{FF}}_{TD}$ represents the feedforward network with target distribution training as the ingredient decoder, while ${\mathrm{TF}}_{set}$ denotes the set transformer model as the ingredient decoder. The set transformer employed explicit cardinality loss to reduce cardinality errors.

Our models generally exhibited inverse correlation between cardinality error and F1 score, with EfficientNetV2-S showing higher error than expected. Despite not employing explicit cardinality loss functions, our approach achieved comparable or superior cardinality accuracy to ${\mathrm{TF}}_{set}$. This result suggests that additional cardinality constraints are unnecessary. Moreover, introducing separate cardinality objectives would likely compromise F1 score optimization, as the primary objective would no longer focus solely on F1 performance. Intuitively, overprediction leads to higher Rc but lower Pr, and vice versa for underprediction. As the F1 score is the harmonic mean of Pr and Rc, the F1 score would be closer to the lower of these two metrics. Therefore, to achieve optimal F1 score, the model needs to balance Pr and Rc. The model is thus encouraged to avoid overprediction and underprediction, resulting in low cardinality error.

#### 4.4.3. Individual Ingredient Performance Distribution

**Figure 4:**
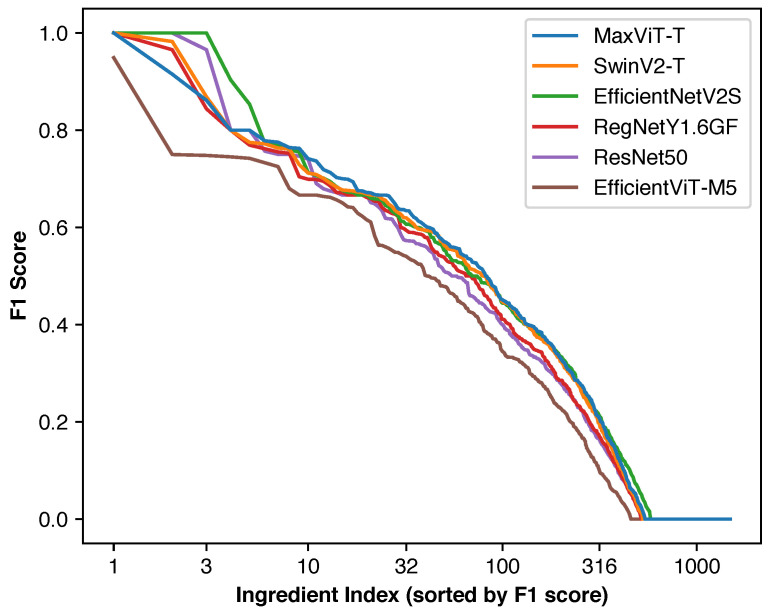
Distribution of F1 scores for individual ingredients when optimizing for micro F1 score for each model. Most rare ingredients have F1 scores of zero as they are difficult to predict due to the lack of training images and their contribution to the overall F1 score is extremely small.

Figure 4 illustrates the distribution of individual ingredient F1 scores across different deep learning backbones. Since ingredient ordering varies between backbones and micro F1 score is not a simple average of individual scores, direct inference of overall performance from these curves is not possible. Nevertheless, EfficientViT-M5 showed consistently lower performance across ingredients, aligning with previous results.

Given that 684 of 1488 ingredients have 50 or fewer training images and rare ingredients minimally impact overall micro F1 scores, 910–1030 ingredients having zero F1 scores is expected. The few ingredients with perfect F1 scores typically have very low frequencies, contributing minimally to overall performance.

### 4.5. F-Score Optimization Method Comparison for Micro F1 Score

To illustrate how much of the F1 score improvements over previous work were due to the Fola algorithm rather than the deep learning models or the overall architecture, we performed an ablation study using MaxViT-T. We compared our approach against soft $F_{\beta }$ loss and BCE loss with fixed $w_{\mathrm{pos}}$ values. Using our Fola algorithm, the computed optimal $w_{\mathrm{pos}}$ was 2.6. For BCE with fixed $w_{\mathrm{pos}}$, we used logarithmically spaced $w_{\mathrm{pos}}$ values to assess their impact on performance. When the Fola algorithm was used, $w_{\mathrm{pos}}$ was adjusted only during validation-based hyperparameter tuning, making results equivalent to BCE with fixed $w_{\mathrm{pos}}=2.6$. Results are presented in Table 6.

Our Fola approach achieved the highest IoU and F1 scores. Soft $F_{\beta }$ loss’s performance was 3.43 and 3.11 percentage points lower in IoU and F1 score, respectively, compared to our method. The differences in F1 scores between Fola and all other approaches were statistically significant (*p*-value ≤0.0027). This demonstrates that while direct F-score optimization using continuous F-score is feasible, our cost-sensitive approach achieves superior performance.

Varying $w_{\mathrm{pos}}$ directly controls the Pr-Rc tradeoff. Higher values prioritize the positive class, yielding higher Rc but lower Pr. Deviating from the optimal $w_{\mathrm{pos}}$ value identified by our algorithm consistently reduced F1 scores. Larger deviations caused greater performance degradation, as expected. These results demonstrate that optimizing $w_{\mathrm{pos}}$ through trial-and-error requires time-consuming, fine-grained search procedures.

### 4.6. Model Comparison for Macro F1 Score

#### 4.6.1. Overall Prediction Performance

To demonstrate our method’s effectiveness for macro F-score optimization, we conducted similar experiments using macro F1 score as the primary metric. Macro F1 score computation involves first calculating individual ingredient F1 scores. The macro F1 score is the unweighted average of these individual scores. This metric ensures equal contribution from each ingredient.

An issue arises when using the macro F1 score. Rare ingredients are associated with few training images. Standard deep learning models do not generalize well in such cases. Instead of being a meaningful measure of accuracy, the F1 scores of these ingredients would be no more than random noise. If the rare ingredients are included, the high variance of this noise would eclipse the small differences between F1 scores of ingredients with sufficient images. Our main objective is to compare and rank different methods, rather than to accurately measure individual F1 scores. This filtering step is necessary to achieve this objective. Therefore, we included only ingredients with at least 1000 images, yielding 314 ingredients after filtering. This large ingredient set is still realistic enough to be used in use cases such as nutrition estimation, because it represents the most commonly used ingredients.

Images containing none of the remaining 314 ingredients were removed from the dataset. Within this set of filtered ingredients, the class imbalance was still severe. The overall negative-to-positive ratio was 39:1, and the class sizes ranged from 1000 to 361,020. For this experiment, we used the Fola algorithm with a bounding factor of 2 to maximize classification performance. Results are presented in Table 7.

Model rankings and relative performance patterns mirror those from micro F1 score experiments. However, macro F1 scores are substantially lower than micro F1 scores, as expected when all ingredients receive equal importance. The median number of positive training images per ingredient was only 4119, compared to 94,961 for the 16th most common ingredient, which was effectively the median ingredient in micro F1 experiments. Nevertheless, MaxViT-T achieved a respectable macro F1 score of 0.3390. All models exhibited higher Rc than Pr, with slight variations in Pr-Rc balance across architectures. The differences in F1 scores between MaxViT-T and the other five models were statistically significant (*p*-value ≤0.0023).

The lower absolute macro F1 scores compared to micro F1 scores were simply caused by the different weighting methods. Actual changes in individual ingredients’ F1 scores due to the equal focus given to each ingredient were tiny in comparison. More details are given in Section 4.6.4.

#### 4.6.2. Cardinality Analysis

Table 8 presents cardinality results for macro F1 score optimization. Optimizing for macro F1 score forced the models to emphasize less common ingredients, resulting in predicted ingredient sets larger than ground truth across all models. The intuitive explanation for this effect is as follows. Underprediction for rare ingredients is likely to lead to F1 scores of zero, as TP = 0. On the other hand, overprediction leads to positive (but low) F1 scores, as both Pr and Rc are nonzero. Therefore, all models erred on the side of overprediction instead of underprediction. A weak inverse correlation exists between cardinality error and F1 score, though EfficientNetV2-S again showed higher error than expected. These results suggest macro F1 score optimization leads to ingredient overprediction. For applications requiring low cardinality error, micro F1 score optimization or explicit cardinality constraints would be preferable.

#### 4.6.3. Individual Ingredient Performance Distribution

**Figure 5:**
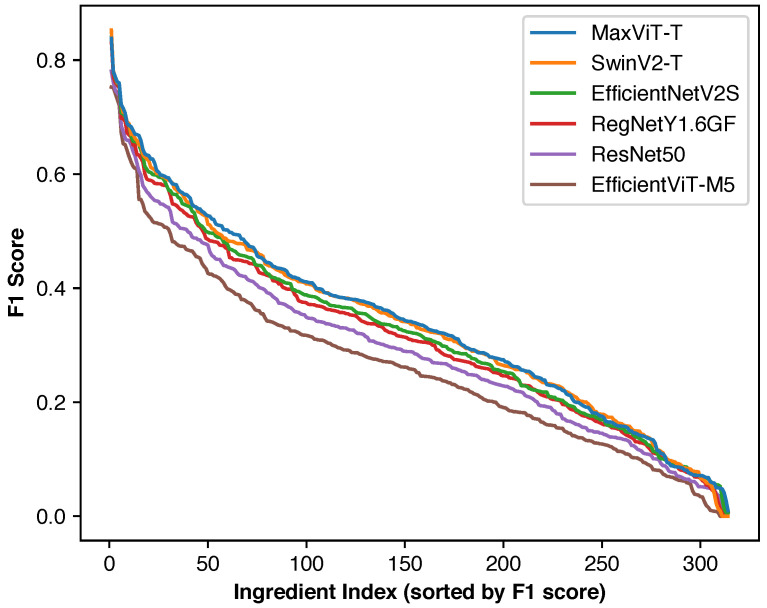
Distribution of F1 scores of individual ingredients when optimizing for macro F1 score for each model. As rare ingredients were excluded, only a small number of ingredients have very small F1 scores.

Figure 5 shows individual ingredient F1 score distributions for macro F1 optimization. Since macro F1 score represents a simple average of individual ingredient F1 scores, overall performance can be directly inferred from these curves. Excluding ingredients with insufficient training data results in fewer ingredients with very low F1 scores (≤0.05). Model rankings remain consistent across most of the performance range.

#### 4.6.4. Micro vs. Macro F1 Score Performance Analysis

**Figure 6:**
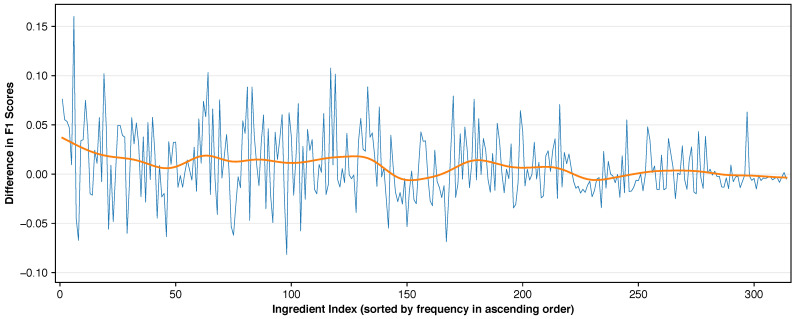
Difference in F1 scores for individual ingredients between micro and macro F1 score optimization (negative values indicate lower macro F1 performance). The orange line represents the Gaussian moving average with a bandwidth of 8. This plot illustrates that when optimizing for the macro F1 score, the model focuses more on the less common ingredients. The effect can be observed as positive moving average values for the first 130 ingredients.

To quantify the differences between micro and macro F1 score optimization, we compared individual ingredient F1 scores for MaxViT-T across both scenarios. The results are depicted in Figure 6. Individual F1 score changes ranged from −0.08 to +0.16, with an average absolute change of 0.026. The general trend showed models slightly sacrificing accuracy on common ingredients to improve performance on rare ingredients. These extensive adjustments across all classes increased overall macro F1 score by 0.0072, from 0.3318 to 0.3390. These results demonstrate the Fola algorithm’s effectiveness for optimizing both micro and macro F1 scores.

#### 4.6.5. Ingredient Frequency vs. Prediction Accuracy

**Figure 7:**
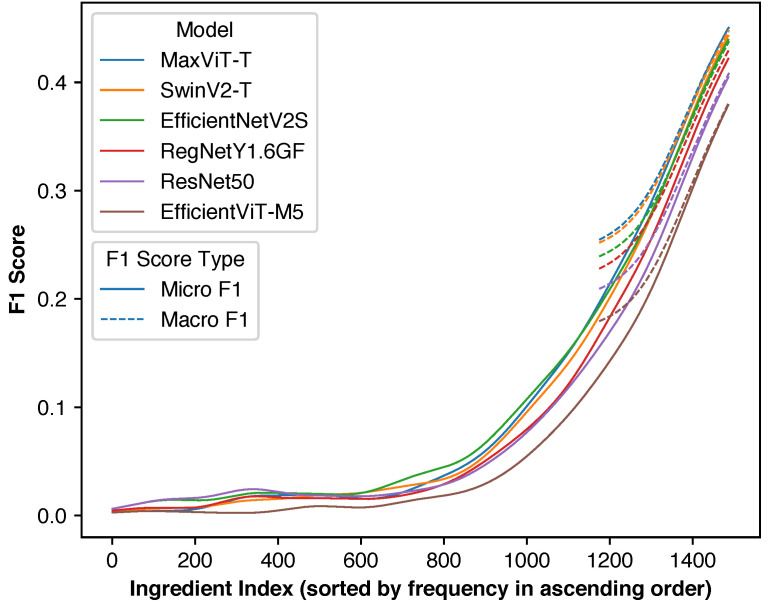
Relationship between ingredient frequency and F1 score when optimizing for micro (solid lines) and macro (dashed lines) F1 scores. Curves are smoothed using Gaussian moving averages with a bandwidth of 74.4. This plot illustrates that by changing from the micro F1 score to the macro F1 score, the F1 scores of the least common ingredients significantly increase.

Figure 7 illustrates how ingredient frequency affects individual F1 scores across all models for both optimization scenarios. Ingredients are sorted by ascending frequency. The curves were smoothed using Gaussian moving average with a bandwidth of 74.4.

For micro F1 score optimization, all six models followed similar trends where the least common ∼900 ingredients had low F1 scores, and performance increased with frequency for the remaining ∼600 ingredients. EfficientNetV2-S performed well for mid-range frequency ingredients but slightly underperformed MaxViT-T and SwinV2-T on the most common ingredients. Since micro F1 score weights ingredients by frequency, EfficientNetV2-S remained behind the top performers in overall micro F1 score.

For macro F1 score optimization, only the top 314 ingredients were retained, but the curves remain directly comparable to micro F1 results. F1 scores for common ingredients were virtually unchanged from micro F1 optimization. On the contrary, significant improvements can be observed for less common ingredients. These results illustrate how changing the objective function shifts model focus and affects classification performance.

**Figure 8:**
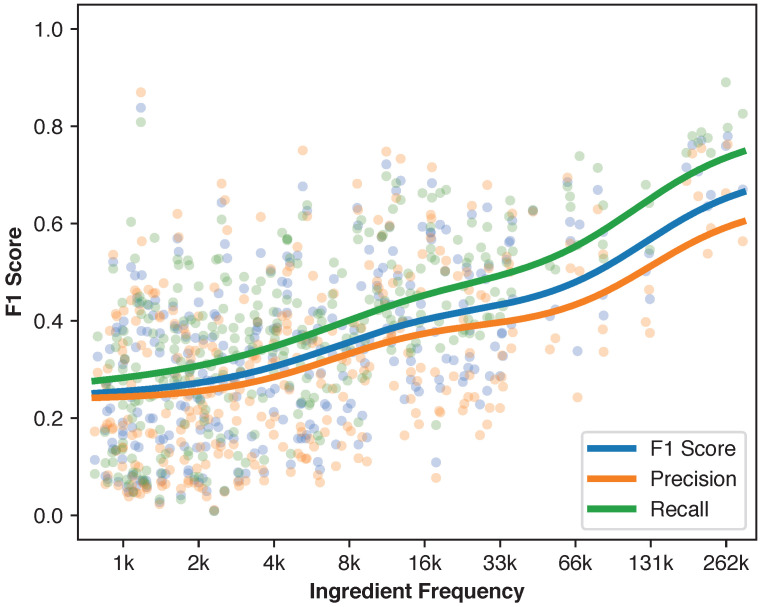
Scatterplot of ingredient frequency versus prediction accuracy for MaxViT-T when optimizing for macro F1 score. Each dot represents an ingredient. The lines are computed using Gaussian moving average with a bandwidth of 1 in logarithm base 2. This plot illustrates how ingredient frequency affects F1 score.

To examine the relationship between ingredient frequency and prediction accuracy more closely, Figure 8 plots each ingredient’s training set frequency against macro F1 score for MaxViT-T. Since all ingredients were equally weighted, differences in individual ingredient F1 scores stemmed from inherent prediction difficulty and training data availability.

Large variance in ingredient prediction difficulty is evident from the scatter points, but clear trends emerge from moving average curves. Less common ingredients tend to have lower Pr and Rc due to insufficient training examples for learning image-ingredient relationships. Both Pr and Rc increase slowly with ingredient frequency. However, after the frequency of approximately 50,000, both metrics increase more rapidly. These results suggest that data availability remains an important bottleneck for visual ingredient prediction, particularly for less commonly used ingredients.

### 4.7. F-Score Optimization Method Comparison for Macro F1 Score

To demonstrate how the Fola algorithm compares to other optimization approaches, we performed an ablation study using MaxViT-T. Using the macro F1 score as the objective function, we again compared our Fola algorithm to soft $F_{\beta }$ loss and BCE loss with fixed $w_{\mathrm{pos}}$. To evaluate the effects of $w_{\mathrm{pos}}$ bounding, we included both bounded (using a bounding factor of 2) and unbounded versions of our algorithm. For BCE with fixed $w_{\mathrm{pos}}$, we used identical values across all ingredients. The varied $w_{\mathrm{pos}}$ values were logarithmically spaced. However, our Fola algorithm computes different optimal $w_{\mathrm{pos}}$ values for each ingredient, making it non-equivalent to fixed-weight BCE. We varied $w_{\mathrm{pos}}$ values to identify optimal settings and assess performance impacts. Results are presented in Table 9.

Reported Pr and Rc values are the average values across all ingredients. Since the macro F1 score is computed from individual F1 scores rather than average Pr and Rc, the overall F1 score may not equal the harmonic mean of average Pr and Rc.

Fola with $w_{\mathrm{pos}}$ bounding achieved the best F1 score, followed closely by BCE loss with fixed $w_{\mathrm{pos}}=5.20$. With fixed $w_{\mathrm{pos}}$, higher $w_{\mathrm{pos}}$ values tended to produce higher Rc and lower Pr. Among the evaluated values, $w_{\mathrm{pos}}=5.20$ yielded optimal F1 performance. Interestingly, lower $w_{\mathrm{pos}}$ values produced only slightly suboptimal F1 scores, while higher values created larger performance gaps. The differences in F1 scores between Fola and all other approaches were statistically significant (*p*-value ≤0.0270).

Soft $F_{\beta }$ loss achieved significantly lower F1 scores than other approaches, though its IoU remained comparable. Since IoU is instance-based, while F1 score is label-based, models with high IoU do not necessarily achieve optimal F1 scores. Upon inspection of training progression across the epochs with the soft $F_{\beta }$ loss, we found that the model initially achieved higher Pr than Rc. As training continued, the model was able to improve the balance between Pr and Rc, resulting in continuously increasing F1 scores. However, both the initial F1 score and the rate of improvement were significantly lower than other approaches. These factors resulted in inferior final F1 scores. Both accuracy and balanced accuracy remain high throughout training at 0.97–0.98, similarly to other methods.

**Figure 9:**
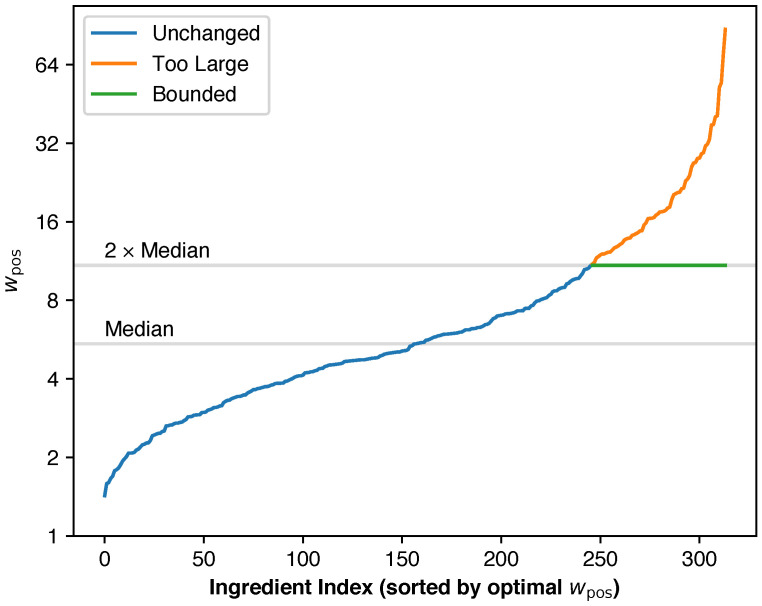
An illustration of how $w_{\mathrm{pos}}$ bounding works. The curves are the real optimal $w_{\mathrm{pos}}$ values for each ingredient before (blue and orange) and after bounding (blue and green), with MaxViT-T when optimizing for macro F1 score. $w_{\mathrm{pos}}$ bounding prevents disproportionately large step sizes which cause overshooting.

When using Fola, $w_{\mathrm{pos}}$ bounding significantly improved the results. A detailed analysis of optimal $w_{\mathrm{pos}}$ values before bounding is necessary to understand why bounding improved the results. Figure 9 illustrates the distribution of optimal $w_{\mathrm{pos}}$ values before bounding. Values ranged from 1.43 to 87.5, with a median of 5.44. $w_{\mathrm{pos}}$ values for 68 ingredients were bounded in total. Bounding $w_{\mathrm{pos}}$ values increased F1 score by 0.0038, suggesting that disproportionately large weight updates from extreme $w_{\mathrm{pos}}$ values degraded model performance.

### 4.8. Discussion

To summarize, within the context of visual food ingredient prediction, our Fola algorithm achieved superior or equivalent F1 scores compared to BCE loss with fixed but properly tuned $w_{\mathrm{pos}}$ for both micro and macro F1 score optimization. Our approach offers greater computational efficiency; only a single validation run and a test run are required due to the absence of hyperparameters that require tuning. The soft $F_{\beta }$ loss alternative did not perform as well as either BCE-based approach.

When compared to existing methods for visual food ingredient prediction, our approach significantly outperformed state-of-the-art results while using comparable or smaller image encoding backbones. Among the six backbone architectures evaluated, MaxViT-T consistently produced the best results across all experimental conditions.

While our work establishes new performance benchmarks for visual food ingredient prediction and provides a principled framework for F-score optimization, several limitations warrant discussion. For practical applications in nutrient monitoring and dietary assessment, ingredient identification alone is insufficient for accurate nutrient estimation. Comprehensive nutritional analysis requires additional information including food weight or volume, preparation methods (e.g., fried, boiled, raw), and relative proportions of each ingredient.

Visual ingredient prediction already presents substantial challenges, as evidenced by the modest absolute F1 scores achieved even with our improved methods. Determining these additional factors from images alone would be even more ambitious and is therefore outside the scope of this work. Future work on food computing could explore multi-task learning frameworks that jointly predict ingredients and their quantities, or incorporate additional modalities such as depth information for volume estimation. As a different direction, our proposed method can be applied to other binary tasks with class imbalance such as disease diagnosis and fraud detection to directly optimize for F-scores.

## 5. Conclusions

This paper presented a novel neural network training framework that allows for direct optimization of the F1 score. This formulation addresses the challenge of finding the optimal tradeoff between the two classes when they are highly imbalanced. Our method provides the theoretically optimal formulation of the class weight parameter to control this balance, together with a practical neural network training algorithm. We applied the method to the visual food ingredient prediction task using a straightforward architecture with a CNN or ViT backbone as an image encoder. Small and efficient architectures were used to ensure that they can be used on mobile devices with low inference latency. The image embeddings were directly connected to the output without introducing additional complexity. The experiments demonstrated that our approach significantly outperformed state-of-the-art ingredient prediction methods. Our optimization framework replaces computationally intensive and suboptimal alternatives such as class weight hyperparameter search, soft $F_{\beta }$ loss, and post hoc threshold tuning. Our method can be applied to any binary task in any domain with single or multiple outputs to optimize for either the micro or macro F-scores. Furthermore, our analysis framework and practical algorithm can be applied to other objective functions based on TP, FP, FN, and true negative (TN) as well.

## Figures and Tables

**Table 1:** CNN and ViT backbones used in our work. GFLOPS stands for giga floating point operations per second and is a measure of computational cost for processing an image (lower is better).

Architecture	Parameters	GFLOPS
MaxViT-T [70]	30.9 M	5.56
SwinV2-T [71]	28.4 M	5.94
EfficientNetV2-S [72]	20.3 M	8.37
RegNetY-1.6GF [73]	9.9 M	1.61
ResNet-50 [74]	23.6 M	4.09
EfficientViT-M5 [75]	12.4 M	0.52

**Table 2:** Top 30 ingredients in the Recipe1M dataset.

Number	Ingredient	Frequency	Number	Ingredient	Frequency
1	Salt	361,020	16	Tomato	94,961
2	Sugar	312,773	17	Baking Powder	80,253
3	Pepper	308,828	18	Garlic	79,688
4	Butter	269,332	19	Cinnamon	73,871
5	Cooking Oil	259,742	20	Baking Soda	73,540
6	Egg	245,942	21	Chicken	72,885
7	Onion	239,661	22	Vinegar	64,648
8	Flour	227,431	23	Parsley	61,112
9	Cheese	212,610	24	Potato	52,350
10	Milk	152,686	25	Broth	44,583
11	Water	151,490	26	Carrot	43,953
12	Garlic Cloves	146,192	27	Basil	42,752
13	Juice	99,735	28	Beef	41,401
14	Cream	99,307	29	Soy Sauce	41,373
15	Flavor Extract	99,206	30	Chips	40,015

**Table 3:** Transfer learning and fine-tuning hyperparameters for all models.

Hyperparameter	Value
Batch Size	512
Optimizer	AdamW
Momentum	None
Weight Decay	0.001
LR Schedule	CLR triangular policy [77] with initial LR of 0.05 of maximum LR
Training Length	20 epochs
Data Augmentation	Random horizontal flip
Dropout Rate	0.2
Label Smoothing	None

**Table 4:** Comparison of deep learning backbones and ingredient prediction approaches when optimizing for micro F1 score. Pr and Rc for previous work are not available.

Model	IoU	F1 Score	Pr	Rc
InverseCooking	0.3211	0.4861	-	-
**FIRE**	0.3259	0.4927	-	-
MaxViT-T	**0.4014 ± 0.0003**	**0.5616 ± 0.0006**	0.5408 ± 0.0043	0.5841 ± 0.0040
SwinV2-T	0.3988 ± 0.0016	0.5593 ± 0.0013	0.5366 ± 0.0020	0.5842 ± 0.0034
EfficientNetV2-S	0.3931 ± 0.0006	0.5522 ± 0.0007	0.5171 ± 0.0014	0.5924 ± 0.0008
RegNetY-1.6GF	0.3856 ± 0.0005	0.5454 ± 0.0002	0.5257 ± 0.0006	0.5667 ± 0.0011
ResNet-50	0.3693 ± 0.0013	0.5284 ± 0.0018	0.5083 ± 0.0011	0.5503 ± 0.0049
EfficientViT-M5	0.3552 ± 0.0045	0.5120 ± 0.0057	0.4874 ± 0.0088	0.5392 ± 0.0021

**Table 5:** Cardinality of predicted ingredient sets when optimizing for micro F1 score.

Model	Cardinality	MAE	F1 Score
Ground Truth	8.03 ± 3.09	-	-
${\mathrm{FF}}_{TD}$	8.02 ± 3.24	3.02 ± 2.50	0.4594
${\mathrm{TF}}_{set}$	9.43 ± 2.35	2.56 ± 1.93	0.4861
MaxViT-T	8.68 ± 3.00	2.42 ± 2.03	0.5620
SwinV2-T	8.66 ± 2.95	2.42 ± 2.02	0.5595
EfficientNetV2-S	9.23 ± 3.09	2.61 ± 2.14	0.5514
RegNetY-1.6GF	8.66 ± 3.02	2.48 ± 2.07	0.5456
ResNet-50	8.80 ± 3.14	2.61 ± 2.16	0.5305
EfficientViT-M5	8.73 ± 2.97	2.57 ± 2.12	0.5186

**Table 6:** Comparison of loss functions for micro F1 score optimization with MaxViT-T model.

Loss Function	IoU	F1 Score	Pr	Rc
soft $F_{\beta }$	0.3671 ± 0.0025	0.5305 ± 0.0027	0.5560 ± 0.0005	0.5073 ± 0.0045
BCE with $w_{\mathrm{pos}}=1.30$	0.3786 ± 0.0015	0.5394 ± 0.0005	0.6185 ± 0.0106	0.4783 ± 0.0070
BCE with $w_{\mathrm{pos}}=1.84$	0.3960 ± 0.0005	0.5574 ± 0.0003	0.5904 ± 0.0033	0.5279 ± 0.0032
Fola ($w_{\mathrm{pos}}=2.60$)	**0.4014 ± 0.0003**	**0.5616 ± 0.0006**	0.5408 ± 0.0043	0.5841 ± 0.0040
BCE with $w_{\mathrm{pos}}=3.68$	0.3949 ± 0.0016	0.5536 ± 0.0015	0.4905 ± 0.0031	0.6352 ± 0.0023
BCE with $w_{\mathrm{pos}}=5.20$	0.3761 ± 0.0042	0.5309 ± 0.0034	0.4387 ± 0.0106	0.6727 ± 0.0145

**Table 7:** Comparison of deep learning backbones and ingredient prediction approaches when optimizing for macro F1 score.

Model	IoU	F1 Score	Pr	Rc
MaxViT-T	**0.3717 ± 0.0012**	**0.3390 ± 0.0009**	0.3123 ± 0.0040	0.3863 ± 0.0092
SwinV2-T	0.3696 ± 0.0005	0.3357 ± 0.0009	0.2998 ± 0.0018	0.4008 ± 0.0030
EfficientNetV2-S	0.3611 ± 0.0007	0.3245 ± 0.0005	0.2812 ± 0.0007	0.4013 ± 0.0014
RegNetY-1.6GF	0.3597 ± 0.0004	0.3164 ± 0.0012	0.2930 ± 0.0021	0.3592 ± 0.0005
ResNet-50	0.3432 ± 0.0002	0.2935 ± 0.0009	0.2746 ± 0.0021	0.3301 ± 0.0032
EfficientViT-M5	0.3253 ± 0.0002	0.2653 ± 0.0006	0.2381 ± 0.0023	0.3141 ± 0.0040

**Table 8:** Cardinality of predicted ingredient sets when optimizing for macro F1 score.

Model	Cardinality	MAE	F1 Score
Ground Truth	7.85 ± 3.08	-	-
MaxViT-T	10.41 ± 4.06	3.51 ± 2.84	0.3399
SwinV2-T	10.94 ± 4.13	3.84 ± 3.02	0.3367
EfficientNetV2-S	11.30 ± 4.30	4.11 ± 3.21	0.3241
RegNetY-1.6GF	10.55 ± 4.12	3.65 ± 2.97	0.3156
ResNet-50	10.68 ± 4.24	3.86 ± 3.09	0.2944
EfficientViT-M5	11.00 ± 4.40	4.10 ± 3.27	0.2651

**Table 9:** Comparison of loss functions for macro F1 score optimization with MaxViT-T model.

Loss Function	IoU	F1 Score	Pr	Rc
soft $F_{\beta }$	0.3736 ± 0.0024	0.1538 ± 0.0012	0.1615 ± 0.0052	0.1706 ± 0.0041
Fola (unbounded)	0.3668 ± 0.0026	0.3352 ± 0.0012	0.3076 ± 0.0021	0.3865 ± 0.0031
Fola (bounded)	0.3717 ± 0.0012	**0.3390 ± 0.0009**	0.3123 ± 0.0040	0.3863 ± 0.0092
BCE with $w_{\mathrm{pos}}=1.84$	0.3963 ± 0.0003	0.3260 ± 0.0015	0.4166 ± 0.0025	0.2871 ± 0.0028
BCE with $w_{\mathrm{pos}}=2.60$	**0.4000 ± 0.0002**	0.3368 ± 0.0014	0.3813 ± 0.0020	0.3193 ± 0.0025
BCE with $w_{\mathrm{pos}}=3.68$	0.3985 ± 0.0003	0.3362 ± 0.0005	0.3521 ± 0.0043	0.3497 ± 0.0037
BCE with $w_{\mathrm{pos}}=5.20$	0.3831 ± 0.0011	0.3373 ± 0.0006	0.3107 ± 0.0045	0.3962 ± 0.0052
BCE with $w_{\mathrm{pos}}=7.35$	0.3677 ± 0.0015	0.3336 ± 0.0016	0.2792 ± 0.0035	0.4287 ± 0.0035
BCE with $w_{\mathrm{pos}}=10.40$	0.3612 ± 0.0015	0.3241 ± 0.0015	0.2745 ± 0.0023	0.4097 ± 0.0012

## Data Availability

The original contributions presented in the study are included in the article, further inquiries can be directed to the corresponding author.

## Abbreviations

The following abbreviations are used in this manuscript:

CNN	Convolutional neural network
ViT	Vision transformer
Pr	Precision
Rc	Recall
BCE	Binary cross-entropy
SGD	Stochastic gradient descent
TP	True positive
FP	False positive
FN	False negative
M	Million
FC	Fully connected
GFLOPS	Giga floating point operations per second
MAE	Mean absolute error
CLR	Cyclical learning rates
LR	Learning rates
IoU	Intersection over union
TN	True negative
